# Influences of probiotics plus enteral nutrition on intestinal flora and inflammatory response in patients with severe acute pancreatitis

**DOI:** 10.3389/fmed.2026.1766361

**Published:** 2026-03-09

**Authors:** Liping Cheng, Chunying Jiang, Wei Jiang, Changhong Zhu

**Affiliations:** 1 Department of Gastroenterology, Changzhou Cancer Hospital, Changzhou, China; 2 Department of Hepatobiliary Surgery, Changzhou Cancer Hospital, Changzhou, China

**Keywords:** enteral nutrition, inflammatory response, intestinal flora, probiotics, severe acute pancreatitis

## Abstract

**Introduction:**

Severe acute pancreatitis (SAP) is frequently accompanied by intestinal barrier disruption, systemic inflammation, and gut microbiota dysbiosis. Although enteral nutrition (EN) is standard in SAP management, EN alone may not fully restore the intestinal microenvironment. This study evaluated whether probiotics combined with EN improve intestinal mucosal integrity, inflammatory responses, immune status, nutritional indices, and intestinal flora in SAP patients.

**Methods:**

In this prospective, randomized, double-blind, placebo-controlled trial, 130 SAP patients were assigned to receive EN plus probiotics or EN plus placebo for 14 days. All patients received standardized supportive care and post-pyloric EN. Outcomes included time to abdominal pain relief, hospital stay, mortality, persistent organ failure, ICU utilization, intestinal mucosal barrier markers (endotoxin, diamine oxidase), inflammatory cytokines (CRP, TNF-α, IL-6), immune indicators (IgG, IgM, IgA), nutritional markers (prealbumin, albumin, transferrin), and quantitative fecal microbiota counts at baseline, day 7, and day 14. Statistical significance was defined as *p* < 0.05.

**Results:**

Compared with controls, the probiotic group showed shorter abdominal pain relief time (4.21 ± 0.53 vs. 5.85 ± 0.62 days) and reduced hospital stay (20.02 ± 2.14 vs. 26.34 ± 3.25 days) (both *p* < 0.05). No differences were found in 28-day mortality, in-hospital mortality, persistent organ failure, ICU admission, or ICU stay. Probiotics produced greater reductions in endotoxin, diamine oxidase, CRP, TNF-α, and IL-6, and greater increases in IgG, IgM, IgA, prealbumin, albumin, and transferrin. The study group also exhibited higher lactobacilli and bifidobacteria counts and lower enterobacteria and enterococci counts at days 7 and 14 (all *p* < 0.05).

**Discussion:**

Probiotics combined with EN improved intestinal barrier function, inflammation, immunity, nutrition, and microbiota composition, although without reducing mortality or major complications.

## 1 Introduction

Acute pancreatitis (AP) is a common acute abdominal condition, most frequently triggered by biliary tract disease, alcohol consumption, or hyperlipidemia, leading to the premature activation of pancreatic enzymes (1). The hallmark of AP is a localized pancreatic inflammatory response, which can progress to edema, hemorrhage, and necrosis (2). This local injury drives the release of cytokines and inflammatory mediators, potentially resulting in systemic inflammatory response syndrome (SIRS) and multiple organ failure (3).

Clinical severity classifies AP as mild (MAP) or severe (SAP). SAP is characterized by a rapid onset, aggressive progression, and high mortality (4). Patients with SAP experience a hypercatabolic and hypermetabolic state, often accompanied by gastrointestinal dysfunction. This contributes to intestinal dysbiosis and bacterial translocation (5). The ensuing systemic inflammatory cascade can alter intestinal microcirculation, leading to mucosal atrophy, increased permeability, and heightened risk of systemic infection (6). Consequently, restoring gut microbiota stability and preserving intestinal barrier function are critical therapeutic goals in SAP management.

Enteral nutrition is a cornerstone of SAP treatment (7). By providing luminal nutrition, enteral nutrition helps maintain intestinal mucosal structure, supports barrier function, and reduces bacterial translocation (8). However, enteral nutrition alone may be insufficient to fully correct the profound dysbiosis and physiological disruptions in SAP (9).

Probiotics, defined as live microorganisms that confer a health benefit when administered in adequate amounts, offer a potential adjunctive therapy (10). Proposed mechanisms include modulating gut microbiota composition, reducing intestinal permeability, promoting mucosal repair, dampening inflammatory responses, and enhancing immune function (10, 11). Their application as an adjunct to enteral nutrition in SAP has garnered increasing interest (12).

Therefore, this study was designed to evaluate the effects of probiotic supplementation combined with standard enteral nutrition on intestinal microbiota and inflammatory responses in patients with SAP.

## 2 Materials and methods

### 2.1 Study design and participants

This single-center, prospective, randomized controlled trial was approved by the hospital’s Ethics Committee. Written informed consent was obtained from all participants or their legally authorized representatives. The clinical trial registration number was ChiCTR2100052968.

**Figure 1:**
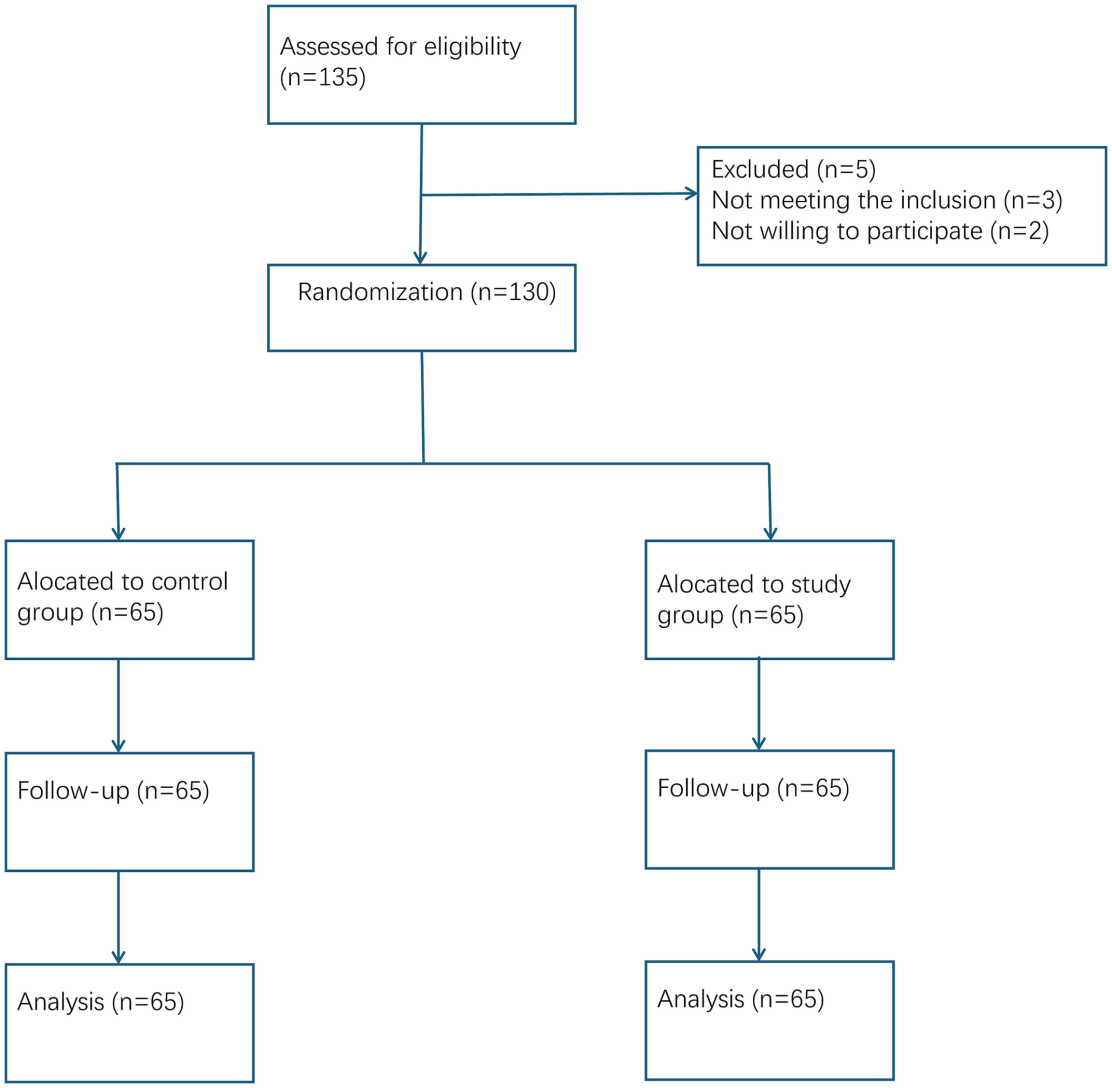
CONSORT flow diagram.

The study enrolled 130 patients with SAP admitted between January 2023 and December 2024. The primary endpoint was defined *a priori* as the length of hospital stay (LOS). The sample size was calculated based on this primary outcome. According to previous studies (13), the mean LOS for SAP patients receiving standard care was estimated to be 28 ± 7 days. We hypothesized that the study intervention could reduce LOS by 4 days. Using a two-sided alpha of 0.05 and a power of 80%, and accounting for an estimated 10% dropout rate, the minimum required sample size was 65 patients per group, totaling 130 patients. The flow diagram is shown in Figure 1.

The diagnosis of SAP was based on the revised Atlanta classification, requiring persistent organ failure (single or multiple) for ≥48 h. Key inclusion criteria were: admission within 48 h of symptom onset; age ≥18 years; and a clinical diagnosis of SAP meeting the revised Atlanta criteria. Exclusion criteria included: mechanical intestinal obstruction; pre-existing severe hepatic, renal, pulmonary, cardiovascular, or cerebrovascular dysfunction; pancreatic malignancy; severe metabolic diseases; pregnancy/lactation; and contraindications to enteral nutrition or probiotics.

### 2.2 Randomization, allocation concealment, and blinding

A double-blind, placebo-controlled design was implemented. The randomization sequence was computer-generated by an independent statistician, who had no involvement in patient recruitment or clinical management, using block randomization (block size of 4). The sequence was stratified based on two key prognostic factors at admission: APACHE II score (<12 vs. ≥12) and Balthazar CT grade (D vs. E). To ensure allocation concealment, the group assignments were placed in sequentially numbered, opaque, sealed envelopes (SNOSE) prepared by the independent statistician. After a participant provided consent, the treating clinician opened the next envelope in sequence to reveal the assigned group. The statistician safeguarded the master randomization list throughout the trial.

A double-blind design was rigorously maintained. Patients were blinded, as both the probiotic and placebo capsules were identical in appearance (size, color), packaging, and administration method. The clinical staff responsible for patient management, the nurses administering the enteral nutrition and study capsules, and the outcome assessors were all blinded to group allocation. The placebo capsules were manufactured specifically for this trial, composed of inert starch, and matched the probiotic capsules in every physical aspect. Outcome assessors for clinical parameters and laboratory technicians performing biochemical analyses were blinded.

### 2.3 Interventions and nutrition protocol

#### 2.3.1 Standard management (both groups)

All patients received standardized supportive care for SAP upon admission. This included continuous monitoring, oxygen therapy, nasogastric tube placement for decompression, fasting, external application of mirabilite, intravenous fluid resuscitation, and electrolyte correction. Human albumin (10 g/day) was supplemented if serum albumin was <30 g/L on admission, until levels reached ≥30 g/L. Parenteral nutrition (PN) was initiated within 24–48 h of admission via central venous access. The PN formulation, providing total daily caloric requirements calculated by the Harris–Benedict formula, consisted of glucose, electrolytes, amino acids (nitrogen source; calorie-to-nitrogen ratio 150:1; insulin-to-glucose ratio 1:4), and fat emulsion (used cautiously in patients with hyperlipidemia).

#### 2.3.2 Criteria and timing for enteral nutrition initiation

Enteral nutrition (EN) was initiated based on standardized clinical criteria, assessed daily by the attending physician blinded to group assignment. The decision required all of the following: (1) improvement in abdominal distension (soft abdomen on palpation), (2) return of bowel sounds, (3) passage of flatus or stool, and (4) no signs of intestinal obstruction.

#### 2.3.3 Enteral nutrition administration protocol (both groups)

Under gastroscopy, a nutritional catheter was inserted into the jejunum, with the tip positioned 25–35 cm distal to the ligament of Treitz. After placement, 500 mL of warm (37–38 °C) normal saline was administered initially. Following gastrointestinal adaptation, the enteral nutrition emulsion (TDF-T, Fresenius Kabi) was delivered as the sole EN formula via a continuous infusion pump. The infusion rate was gradually increased as tolerated until full caloric requirements were met.

#### 2.3.4 Study intervention administration (probiotic/placebo)

##### 2.3.4.1 Product specification

The study group received a commercially available probiotic, Bifid Triple Viable Capsules (Bifico^®^, Shanghai Sine Pharmaceutical Co., Ltd., China; National Drug Approval Number: S10950032; Batch Numbers: 20221205, 20230517). Each capsule (210 mg) contains a lyophilized powder of three live bacterial strains: *Bifidobacterium longum* (CGMCC No. 1.2170), *Lactobacillus acidophilus* (CGMCC No. 1.1854), and *Enterococcus faecalis* (CGMCC No. 1.2131). The manufacturer guarantees a minimum viable count of 1.0 × 10^7^ colony-forming units (CFU) per capsule for each strain, resulting in a total minimum of 3.0 × 10^7^ CFU per capsule. The control group received identically appearing, starch-based placebo capsules manufactured specifically for this trial.

##### 2.3.4.2 Dosing regimen and rationale

The probiotic (or placebo) was delivered via the same nasojejunal tube used for enteral nutrition (positioned 25–35 cm beyond the ligament of Treitz) to ensure post-pyloric delivery. The dosing regimen was adjusted to two capsules, twice daily, based on existing clinical studies supporting this schedule for SAP patients, aiming to enhance bacterial viability and colonization in the lower gastrointestinal tract (14).

Both groups received continuous treatment for 14 days.

### 2.4 Outcome measures

#### 2.4.1 Primary clinical outcome

##### 2.4.1.1 LOS

Calculated from the date of admission to the date of discharge.

##### 2.4.1.2 Time to abdominal pain relief

Defined as the time from admission to a sustained verbal rating score of ≤2 on a 0–10 scale for 24 h.

#### 2.4.2 Secondary clinical outcomes

##### 2.4.2.1 Mortality

28-day all-cause mortality and in-hospital mortality.

##### 2.4.2.2 Organ failure

Incidence and duration (days) of persistent organ failure (respiratory, cardiovascular, renal) as defined by the modified Marshall score.

##### 2.4.2.3 Major infectious complications

Incidence of infected pancreatic necrosis, confirmed by culture of (CT-guided) fine-needle aspiration or operative specimens. Need for intervention (percutaneous drainage or necrosectomy) was recorded.

##### 2.4.2.4 ICU admission & duration

Proportion of patients requiring intensive care unit (ICU) treatment and the length of ICU stay.

#### 2.4.3 Exploratory laboratory and microbiological parameters (assessed at baseline, day 7, and day 14)

##### 2.4.3.1 Intestinal barrier function

Serum endotoxin: Quantified using a chromogenic limulus amebocyte lysate (LAL) assay (Beyotime Biotech Inc., Catalog Number: C0276S). Results are expressed in endotoxin units per milliliter (EU/mL). Serum diamine oxidase (DAO): Measured using a commercially available enzyme-linked immunosorbent assay (ELISA) kit (COIBO BIO, Catalog Number: CB10538-Hu). Concentrations are reported in units per liter (U/L).

##### 2.4.3.2 Systemic inflammation

C-reactive protein (CRP): Measured by immunoturbidimetry (mg/L). Tumor necrosis factor-alpha (TNF-α) and interleukin-6 (IL-6): Quantified using commercially available ELISA kits (COIBO BIO, Catalog Number: CB11762-Hu for TNF-α and Catalog Number: CB10373-Hu for IL-6). Results are expressed in picograms per milliliter (pg/mL).

##### 2.4.3.3 Immune status

Serum levels of immunoglobulins IgA, IgG, and IgM were determined by immunonephelometry (g/L).

##### 2.4.3.4 Nutritional status

Serum concentrations of prealbumin (PA, mg/L), albumin (ALB, g/L), and transferrin (TRF, g/L) were measured using standard biochemical assays.

##### 2.4.3.5 Fecal microbiota composition

Quantitative culture was performed on fresh fecal samples collected at each time point. Within 1 h of collection, 1-gram aliquots were serially diluted and plated on selective media under appropriate conditions: lactobacilli on de Man, Rogosa and Sharpe (MRS) agar (anaerobic, 37 °C, 48 h); bifidobacteria on trypticase-phytone-yeast extract (TPY) agar (anaerobic, 37 °C, 72 h); enterobacteria on MacConkey agar (aerobic, 37 °C, 24 h); and enterococci on bile esculin azide agar (aerobic, 37 °C, 24–48 h). Results are expressed as log₁₀ colony-forming units per gram of feces (log₁₀ CFU/g).

### 2.5 Statistical analysis

Data were analyzed using SPSS software (version 20.0). Continuous variables were assessed for normality using the Shapiro–Wilk test and are reported as mean ± standard deviation (SD). All outcome variables assessed were confirmed to be normally distributed. Between-group comparisons of these variables at each time point were performed using independent samples t-tests. Within-group changes over time were analyzed using repeated-measures analysis of variance (ANOVA), with the assumption of sphericity verified using Mauchly’s test. To control for potential inflation of the type I error rate due to multiple comparisons across variables and time points, the Benjamini–Hochberg false discovery rate (FDR) correction was applied. Effect size was displayed as 95% confidence interval (95% CI). Categorical variables, presented as counts and percentages, were compared between groups using Fisher’s exact test. Statistical significance was defined as a two-tailed *p*-value <0.05.

## 3 Results

### 3.1 General data

No differences were seen in the general data between the two groups (*p* > 0.05, Table 1).

**Table 1:** General data between the two groups.

Items	Control group (*n* = 65)	Study group (*n* = 65)	*p*
Gender, *n* (%)			0.860
Male	35 (53.85)	33 (50.77)	
Female	30 (46.15)	32 (49.23)	
Age (years), mean ± SD	47.98 ± 5.36	48.31 ± 5.43	0.727
APACHE II score (points), mean ± SD	15.42 ± 1.58	15.31 ± 1.52	0.686
Balthazar CT grade, *n* (%)			0.838
Grade D	48 (73.85)	50 (76.92)	
Grade E	17 (26.15)	15 (23.08)	
Etiology of acute pancreatitis, *n* (%)			0.921
Biliary	38 (58.46)	36 (55.38)	
Alcoholic	12 (18.46)	13 (20.00)	
Hypertriglyceridemia	9 (13.85)	10 (15.38)	
Other/Idiopathic	6 (9.23)	6 (9.23)	
Symptom onset to admission (hours), mean ± SD	18.23 ± 6.52	17.80 ± 7.12	0.735
Symptom onset to EN initiation (hours), mean ± SD	92.43 ± 24.71	89.65 ± 22.34	0.496
Symptom onset to study intervention (hours), mean ± SD	92.53 ± 24.65	89.71 ± 22.42	0.488
Organ failure status at randomization, *n* (%)			0.812
Transient organ failure (<48 h)	21 (32.31)	19 (29.23)	
Persistent organ failure (≥48 h)	44 (67.69)	46 (70.77)	

### 3.2 Time to abdominal pain relief and hospital stay

**Figure 2:**
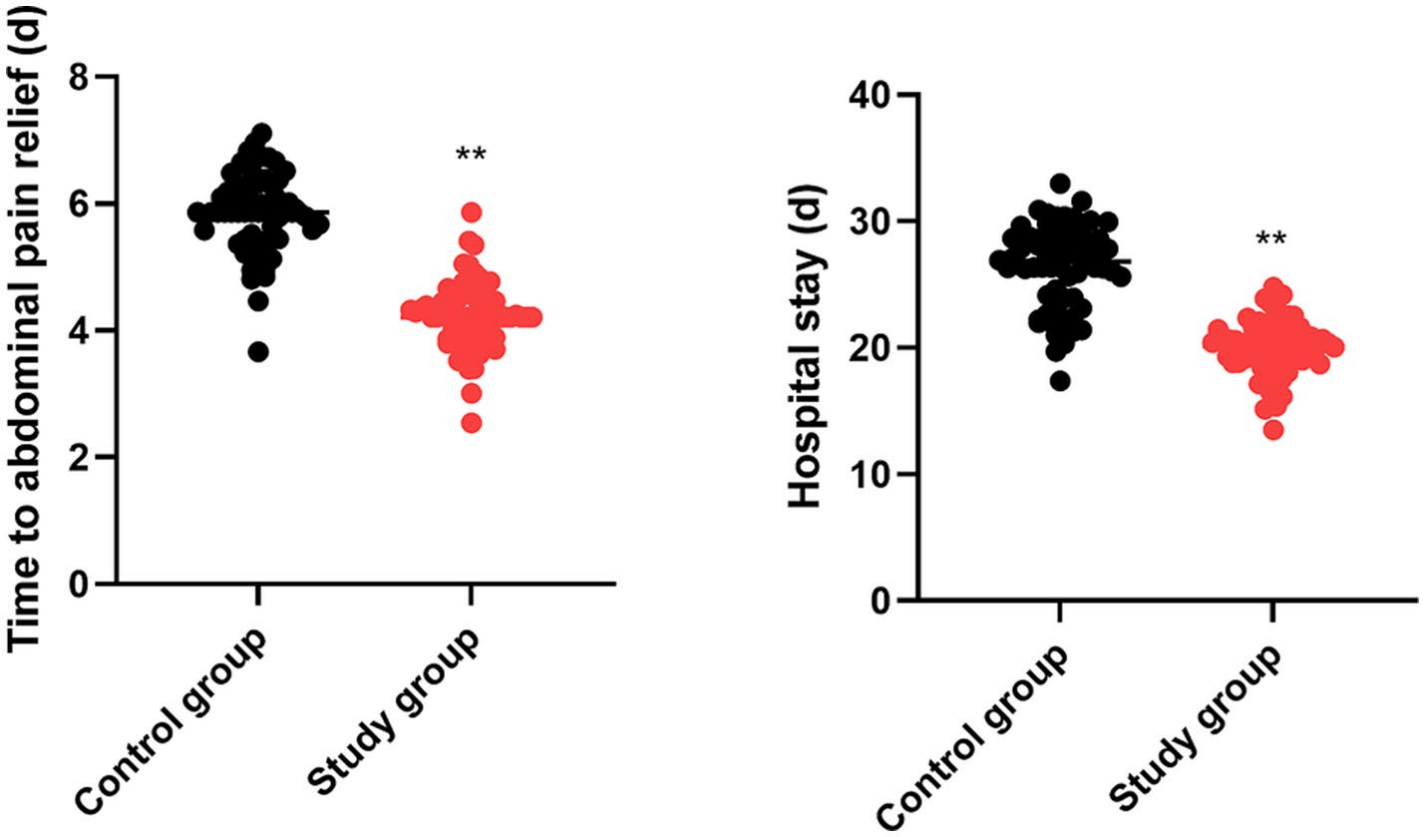
Time for relief of abdominal pain and hospital stay. ^**^p < 0.01.

The time to abdominal pain relief of the control group was (5.85 ± 0.62) days, and that of the study group was (4.21 ± 0.53) days. The hospital stay of the control group was (26.34 ± 3.25) days, and that of the study group was (20.02 ± 2.14) days. Relative to the control group, the study group had shorter time to abdominal pain relief and shorter hospital stay (*p* < 0.05, 95% CI: −1.842 to −1.423; *p* < 0.05, 95% CI: −7.278 to −5.362; Figure 2).

### 3.3 Incidence of mortality

The 28-day all-cause mortality in the study group was 4.62% (3/65), while that in the control group was 6.15% (4/65). The mortality rates during hospitalization in the study group was 6.15% (4/65), while that in the control group was 7.69% (5/65). There were no significant differences in the 28-day all-cause mortality and in-hospital mortality between the two groups (*p* = 0.717 and *p* = 0.731, Table 2).

**Table 2:** Incidence of mortality between the two groups.

Groups	Cases	28-day all-cause mortality	In-hospital mortality
Control group	65	4 (6.15)	5 (7.69)
Study group	65	3 (4.62)	4 (6.15)
*p*		0.717	0.731

### 3.4 Incidence and duration of persistent organ failure

The incidence of persistent organ failure in the study group was 18.46% (12/65), while that in the control group was 23.1% (15/65) in the control group. The duration of organ failure in the study group was (5.58 ± 2.35) days, while that in the control group was (6.08 ± 3.21) days. There were no significant differences in the incidence of persistent organ failure and duration of organ failure between the two groups (*p* = 0.525 and *p* = 0.313, Table 3).

**Table 3:** Incidence and duration of persistent organ failure between the two groups.

Groups	Cases	Incidence of persistent organ failure	Duration of organ failure (days)
Control group	65	15 (23.08)	6.08 ± 3.21
Study group	65	12 (18.46)	5.58 ± 2.35
*p*		0.525	0.312
95% CI		/	−0.477 to 1.477

### 3.5 Proportion requiring ICU care and length of ICU stay

The proportion of patients requiring ICU care in the study group was 43.08% (28/65), while that in the control group was 46.15% (30/65). The length of ICU stay in the study group was (7.53 ± 2.75) days, while that in the control group was (8.05 ± 3.26) days. There were no significant differences in the proportion requiring ICU care and length of ICU stay between the two groups (*p* = 0.722 and *p* = 0.327, Table 4).

**Table 4:** Proportion requiring ICU care and length of ICU stay between the two groups.

Groups	Cases	Proportion requiring ICU care	Length of ICU stay (days)
Control group	65	30 (46.15)	8.05 ± 3.26
Study group	65	28 (43.08)	7.53 ± 2.75
*p*		0.722	0.327
95% CI		/	−0.189 to1.590

### 3.6 Incidence of infected pancreatic necrosis

The incidence of infected pancreatic necrosis in the study group was 9.23% (6/65) of patients in the study group and 13.85% (9/65) in the control group (*p* = 0.416). Of these, four patients in the study group and six patients in the control group required percutaneous drainage or necrosectomy.

### 3.7 Intestinal mucosal barrier indicators

**Figure 3:**
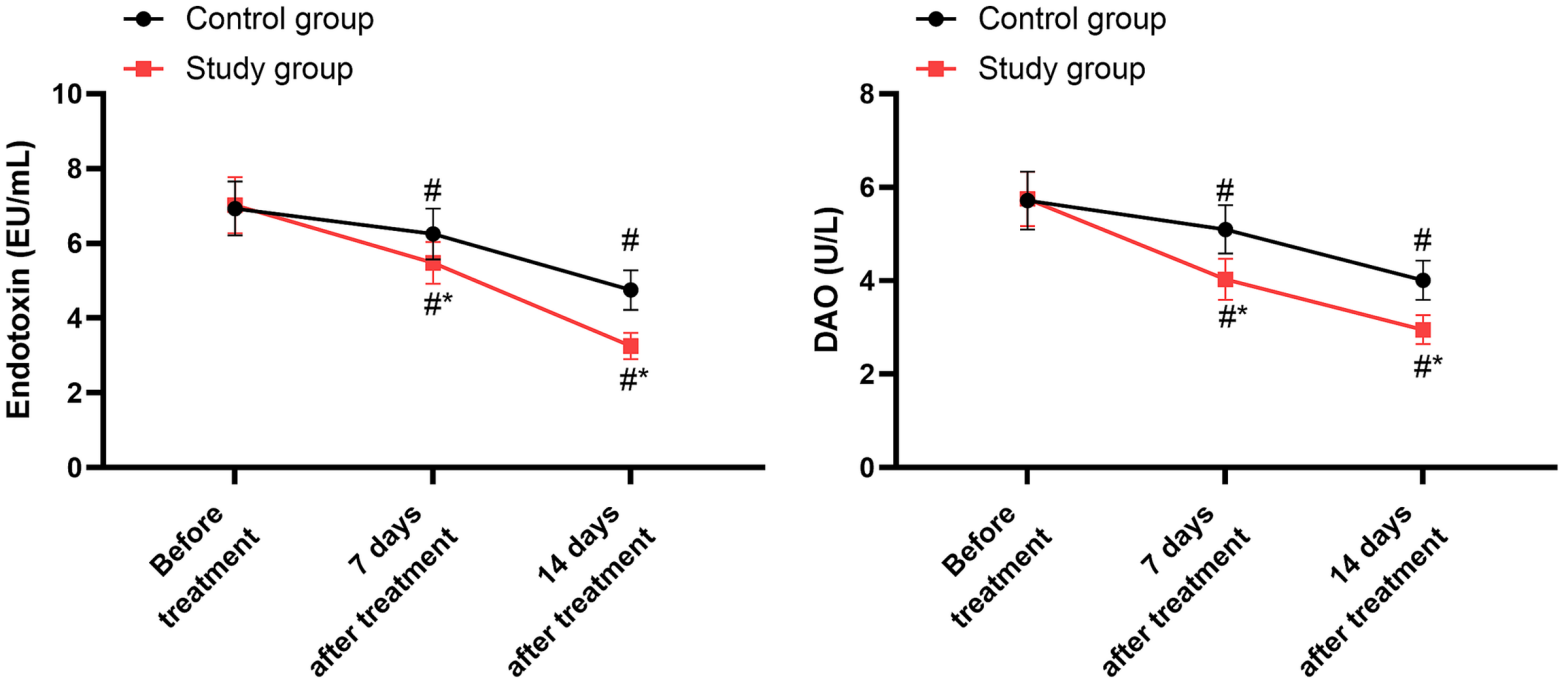
Intestinal mucosal barrier indicators between the two groups. ^#^*p* < 0.05, compared with before treatment; ^*^p < 0.05, compared with the control group.

Prior to the treatment, no significant differences were seen in endotoxin and DAO levels between the two groups (*p* > 0.05). Following 7 days and 14 days of treatment, both groups exhibited a notable decrease in endotoxin and DAO levels compared to their pre-treatment values (*p* < 0.05). When compared to the control group, the study group demonstrated significantly lower levels of endotoxin and DAO on both the 7th and 14th days post-treatment (*p* < 0.05, 95% CI: 0.604–0.848; *p* < 0.05, 95% CI: 0.601–0.798; Figure 3 and Table 5).

**Table 5:** Serum endotoxin and DAO levels at baseline, day 7, and day 14.

Parameter	Group	Baseline	Day 7	Day 14
Endotoxin (EU/mL)	Control group	6.93 ± 0.72	6.25 ± 0.68	4.75 ± 0.53
	Study group	7.02 ± 0.75	5.48 ± 0.56	3.25 ± 0.35
DAO (U/L)	Control group	5.72 ± 0.62	5.10 ± 0.52	4.01 ± 0.42
	Study group	5.75 ± 0.58	4.03 ± 0.44	2.95 ± 0.31

### 3.8 Levels of inflammatory factors

**Figure 4:**
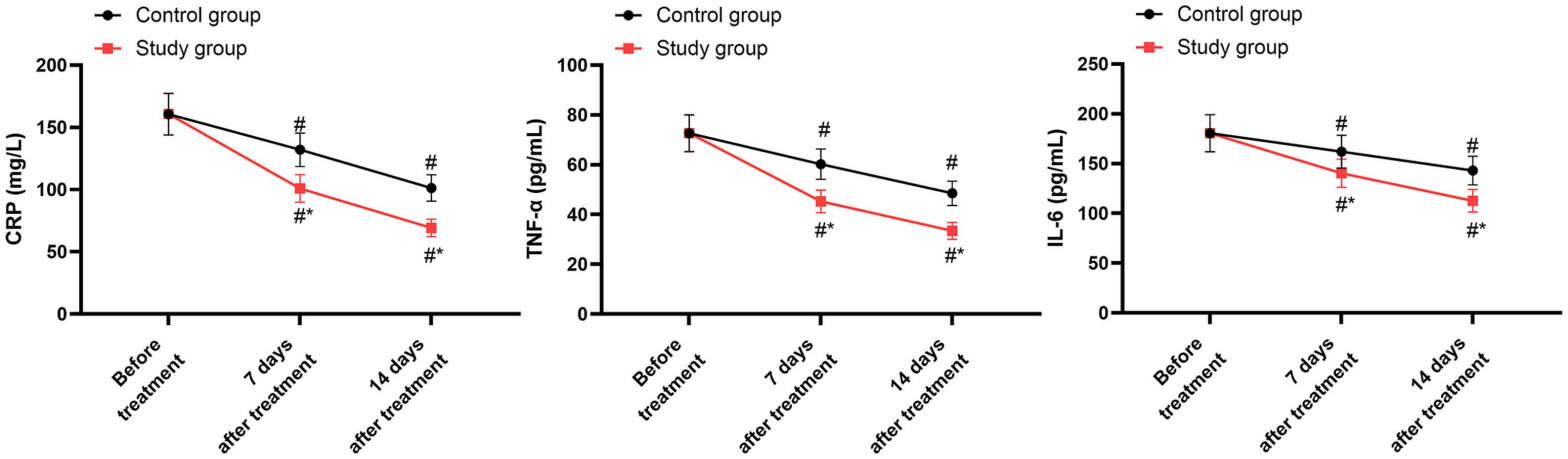
Levels of inflammatory factors between the two groups. ^#^*p* < 0.05, compared with before treatment; ^*^p < 0.05, compared with the control group.

Prior to initiating the treatment, no differences were detected in the concentrations of CRP, TNF-α, and IL-6 between the two groups (*p* > 0.05). After 7 days and 14 days of treatment, both groups showed a marked reduction in the levels of CRP, TNF-α, and IL-6 compared to their baseline values (*p* < 0.05). When compared with the control group, the study group exhibited notably lower levels of CRP, TNF-α, and IL-6 on both the 7th and 14th days following treatment (*p* < 0.05, 95% CI: 18.40–23.61; *p* < 0.05, 95% CI: 8.840–11.14; *p* < 0.05, 95% CI: 14.16–20.44; Figure 4 and Table 6).

**Table 6:** Serum levels of inflammatory factors at baseline, day 7, and day 14.

Parameter	Group	Baseline	Day 7	Day 14
CRP (mg/L)	Control group	160.58 ± 16.74	132.02 ± 13.47	101.32 ± 10.58
	Study group	160.67 ± 16.82	100.98 ± 11.04	69.25 ± 7.03
TNF-α (pg/mL)	Control group	72.62 ± 7.38	60.21 ± 6.05	48.52 ± 4.87
	Study group	72.68 ± 7.29	45.25 ± 4.58	33.45 ± 3.37
IL-6 (pg/mL)	Control group	180.45 ± 18.65	162.02 ± 16.47	143.02 ± 14.38
	Study group	180.57 ± 18.39	140.36 ± 14.16	112.65 ± 11.35

### 3.9 Immune function

**Figure 5:**
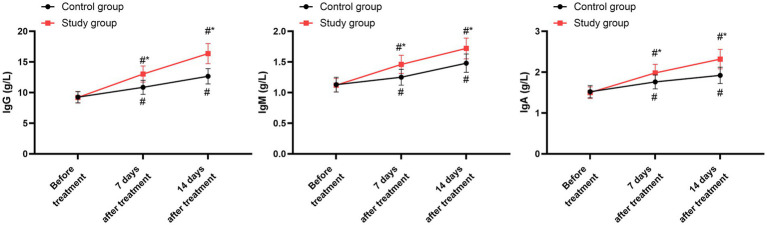
Immune function between the two groups. ^#^*p* < 0.05, compared with before treatment; ^*^p < 0.05, compared with the control group.

Before the commencement of treatment, the two groups exhibited no significant disparities in the levels of IgG, IgM, and IgA (*p* > 0.05). Following 7 days and 14 days of treatment, both groups demonstrated a notable elevation in the levels of IgG, IgM, and IgA compared to their pre-treatment values (*p* < 0.05). When compared with the control group, the study group displayed higher levels of IgG, IgM, and IgA on both the 7th and 14th days post-treatment (*p* < 0.05, 95% CI: −2.188 to −1.699; *p* < 0.05, 95% CI: −0.174 to −0.118; *p* < 0.05, 95% CI: −0.237 to −0.162; Figure 5 and Table 7).

**Table 7:** Serum levels of immune function indicators at baseline, day 7, and day 14.

Parameter	Group	Baseline	Day 7	Day 14
IgG (g/L)	Control group	9.25 ± 0.93	10.85 ± 1.15	12.65 ± 1.25
	Study group	9.21 ± 0.92	13.02 ± 1.32	16.35 ± 1.64
IgM (g/L)	Control group	1.13 ± 0.12	1.25 ± 0.13	1.48 ± 0.15
	Study group	1.12 ± 0.11	1.46 ± 0.15	1.72 ± 0.17
IgA (g/L)	Control group	1.52 ± 0.15	1.76 ± 0.17	1.92 ± 0.20
	Study group	1.50 ± 0.14	1.98 ± 0.21	2.32 ± 0.24

### 3.10 Nutritional status

**Figure 6:**
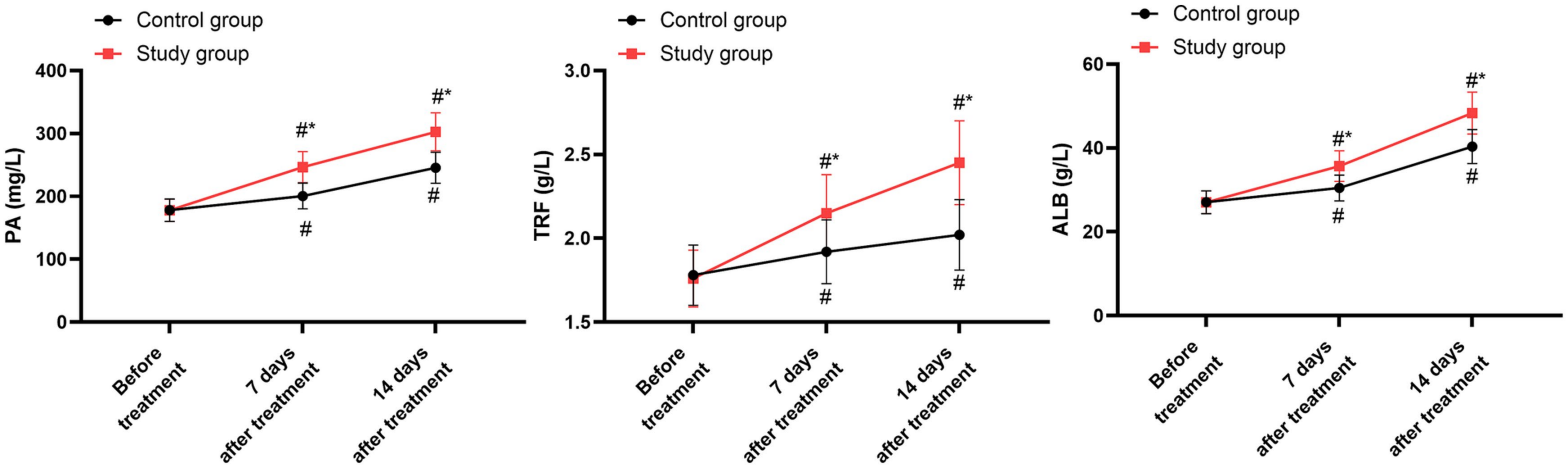
Nutritional status between the two groups. ^#^*p* < 0.05, compared with before treatment; ^*^p < 0.05, compared with the control group.

Prior to the treatment, no significant differences were seen in the concentrations of PA, TRF, and ALB between the two groups (*p* > 0.05). After undergoing 7 days and 14 days of treatment, both groups showed a marked increase in the levels of PA, TRF, and ALB compared to their pre-treatment levels (*p* < 0.05). When compared with the control group, the study group had notably higher levels of PA, TRF, and ALB on both the 7th and 14th days following the treatment (*p* < 0.05, 95% CI: −38.7 to −29.59; *p* < 0.05, 95% CI: −0.254 to −0.172; *p* < 0.05, 95% CI: −5.118 to −3.675; Figure 6 and Table 8).

**Table 8:** Serum levels of nutritional indexes at baseline, day 7, and day 14.

Parameter	Group	Baseline	Day 7	Day 14
PA (mg/L)	Control group	178.02 ± 17.85	200.66 ± 20.65	245.57 ± 24.62
	Study group	177.82 ± 17.62	246.35 ± 24.67	302.62 ± 30.24
TRF (g/L)	Control group	1.78 ± 0.18	1.92 ± 0.19	2.02 ± 0.21
	Study group	1.76 ± 0.17	2.15 ± 0.23	2.45 ± 0.25
ALB (g/L)	Control group	27.05 ± 0.73	30.45 ± 3.05	40.32 ± 4.05
	Study group	27.01 ± 2.0	35.68 ± 3.64	48.32 ± 5.01

### 3.11 Quantity of intestinal flora

**Figure 7:**
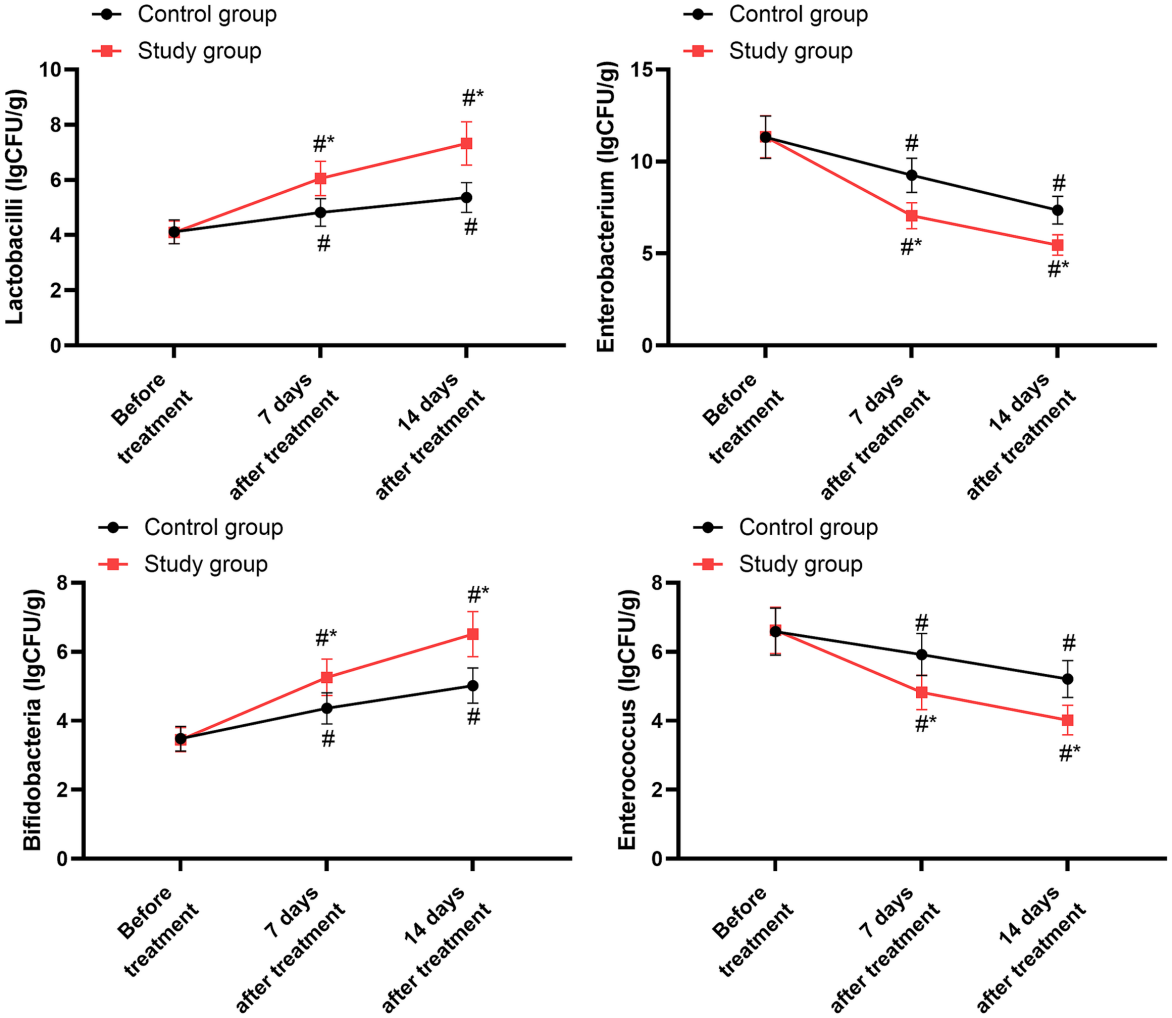
Quantity of intestinal flora between the two groups. ^#^*p* < 0.05, compared with before treatment; ^*^p < 0.05, compared with the control group.

Before initiating the treatment, the two groups showed no significant differences in the counts of lactobacilli, enterobacteria, bifidobacteria, and enterococci (*p* > 0.05). After 7 days and 14 days of treatment, both groups experienced an increase in lactobacilli and bifidobacteria counts compared to their pre-treatment levels (*p* < 0.05), while the counts of enterobacteria and enterococci decreased in both groups (*p* < 0.05). When compared to the control group, the study group exhibited higher lactobacilli counts and lower enterobacteria and enterococci counts on both the 7th and 14th days post-treatment (*p* < 0.05, 95% CI: −1.168 to −0.945; *p* < 0.05, 95% CI: 1.177–1.536; *p* < 0.05, 95% CI: −0.883 to −0.689; *p* < 0.05, 95% CI: 0.635–0.864; Figure 7 and Table 9).

**Table 9:** Quantity of intestinal flora at baseline, day 7, and day 14.

Parameter	Group	Baseline	Day 7	Day 14
Lactobacilli (lgCFU/g)	Control group	4.12 ± 0.43	4.82 ± 0.50	5.36 ± 0.54
	Study group	4.10 ± 0.41	6.05 ± 0.62	7.32 ± 0.78
Enterobacterium (lgCFU/g)	Control group	11.32 ± 1.15	9.25 ± 0.93	7.36 ± 0.75
	Study group	11.35 ± 1.14	7.05 ± 0.71	5.46 ± 0.56
Bifidobacteria (lgCFU/g)	Control group	3.48 ± 0.36	4.36 ± 0.45	5.02 ± 0.51
	Study group	3.45 ± 0.35	5.26 ± 0.53	6.51 ± 0.65
Enterococcus (lgCFU/g)	Control group	6.58 ± 0.68	5.92 ± 0.61	5.21 ± 0.53
	Study group	6.62 ± 0.67	4.82 ± 0.50	4.02 ± 0.43

## 4 Discussion

Enteral nutrition is a cornerstone of management for SAP, providing direct intestinal support that helps maintain mucosal barrier integrity and reduces bacterial or endotoxin translocation (15). However, EN alone may not fully correct the intestinal dysbiosis and impaired motility often present in SAP patients, which supports the rationale for exploring probiotic supplementation (14). Probiotics are proposed to complement EN through several mechanisms: modulating the gut microbiota composition, producing antimicrobial substances (e.g., short-chain fatty acids, bacteriocins), strengthening the mucosal barrier, stimulating digestive enzyme activity, and regulating local and systemic immune responses (16).

This study evaluated the effects of supplemental probiotics alongside standard enteral nutrition in patients with SAP. It is important to emphasize that, consistent with several previous trials and meta-analyses (17), the addition of probiotics in our cohort did not demonstrate a statistically significant impact on primary clinical outcomes, including mortality, persistent organ failure, incidence of infected pancreatic necrosis, or ICU utilization. These findings align with a broader body of evidence suggesting that probiotics may not confer definitive benefits on these critical endpoints in SAP, and notably resonate with the cautionary results of the PROPATRIA trial, which reported increased mortality and intestinal complications in critically ill pancreatitis patients receiving probiotics (18).

However, our exploratory analyses revealed that probiotic supplementation was associated with significant improvements in a range of secondary, surrogate biomarkers. Specifically, the probiotic group exhibited more pronounced reductions in markers of intestinal barrier disruption [endotoxin, diamine oxidase (DAO)] and systemic inflammation [C-reactive protein (CRP), tumor necrosis factor-alpha (TNF-α), interleukin-6 (IL-6)] at days 7 and 14. Similarly, greater improvements were observed in immune parameters (IgG, IgM, IgA) and nutritional markers (prealbumin, transferrin, albumin). Microbiota analysis further indicated a shift towards a potentially more favorable profile, with increased lactobacilli and decreased enterobacteria/enterococci counts in the probiotic group. It is important to note that our analysis utilized traditional culture-based methods. While this approach provides quantitative data for the specific bacterial groups targeted, it has inherent limitations in resolution. Culture-dependent techniques cannot profile uncultivable organisms and offer a less comprehensive view of the entire microbial community compared to molecular methods such as 16S rRNA gene sequencing. Nevertheless, the observed shifts in the cultivable fractions are consistent with the intended action of the probiotic supplement. These observed biochemical and microbial changes are biologically plausible and suggest that probiotics may positively influence surrogate pathways related to gut barrier function, inflammation, and immunity in SAP, which are consistent with previous studies (16, 19). The shorter time to abdominal pain relief and reduced hospital stay in the probiotic group, while noteworthy, derive from secondary analyses and must be interpreted with caution. They hint at a potential modulation of the clinical course but do not equate to a proven effect on hard outcomes.

In conclusion, this study provides exploratory, hypothesis-generating evidence that adjuvant probiotic therapy may improve surrogate markers of intestinal health, inflammation, immunity, and nutrition in SAP patients receiving enteral nutrition. However, these findings must be clearly framed within the context of the neutral primary clinical outcomes and the mixed evidence base, including studies that have raised safety concerns. Our results do not support a definitive clinical benefit for probiotics in SAP regarding mortality or major morbidity. Future large-scale, rigorously designed trials are necessary to determine whether modulating these surrogate pathways can translate into meaningful and safe clinical improvements, and to better identify which patient subgroups, if any, might benefit from such interventions.

## Data Availability

The datasets presented in this study can be found in online repositories. The names of the repository/repositories and accession number(s) can be found in the article/supplementary material.
